# The Use of Semiconductor Quantum Dots with Large, Built-In Spontaneous Polarizations for the Electric Potential Stimulation of Biological Structures on the Nanoscale

**DOI:** 10.3390/nano13243143

**Published:** 2023-12-15

**Authors:** Nida Zia, Michael A. Stroscio, Mitra Dutta

**Affiliations:** 1 Department of Electrical and Computer Engineering, University of Illinois at Chicago, Chicago, IL 60607, USA; nzia3@uic.edu; 2 Richard and Loan Hill Department of Biomedical Engineering, Electrical and Computer Engineering, and Physics, University of Illinois at Chicago, Chicago, IL 60607, USA; 3 Department of Electrical and Computer Engineering and Physics Department, University of Illinois at Chicago, Chicago, IL 60607, USA; dutta@uic.edu

**Keywords:** quantum dot, built-in spontaneous polarization, Debye screening, electric potential

## Abstract

The feasibility of using quantum dots fabricated from materials with built-in spontaneous polarizations for the electric potential stimulation of biological structures in aqueous environments is evaluated by modeling the electric potential produced in the vicinity of such quantum dots. By modeling the external potential created by the spherical nanoscale region of a material with spontaneous polarization, and by considering Debye screening in the vicinity of the quantum dot, it is found that electric potential around these nanostructures is sufficient to cause physiological effects in selected biological systems. These findings suggest that quantum dots may be used in lieu of quantum dots with polarizations produced using an external laser to cause physiological effects. The elimination of the external laser represents a significant benefit of using quantum dots with permanent, built-in spontaneous polarization.

## 1. Introduction

The use of external fields is a dominant approach within the expanding field of bioelectronics. One of the specific approaches in bioelectronics involves the use of patch-clamp techniques to control the opening and closing of voltage-gated ion channels which span the bilipid cellular membranes of cells. Ion channels are protein molecules that span the cell membrane, which is a bilipid layer consisting of phosphate heads on both sides of the cell membrane and fatty acid tails in between [1]. These ion channels can be opened and closed in various ways, for instance, by changing the membrane potential using voltage, binding a ligand, using chemicals, etc. Once open, these selective ion channels allow specific ions to pass through them. As discussed in [1], voltage-gated ion channels can be gated—opened or closed—via the application of a potential of a few mV across them. The external potential in such situations is traditionally applied by using an electrode that is located close to the extracellular region of a voltage-gated ion channel. The use of such an electrode represents a major undertaking and generally involves the meticulous and precise placement of an external electrode close to the extracellular region of a voltage-gated ion channel. The tremendous advances in nanotechnology now make it possible to synthesize spherical nanocrystals, which are suspended in colloidal suspensions. Such colloidal nanocrystals—often referred to as quantum dots—are most frequently composed of materials that do not have built-in external potentials, and these nanostructures have been utilized in many applications since the pioneering studies of [2].

Since the original, pioneering research of [2] has shown that these quantum dots may be synthesized from materials that do have large, internal, built-in spontaneous polarizations. Examples of such synthesis techniques are given in [3,4,5,6].

**Figure 1:**
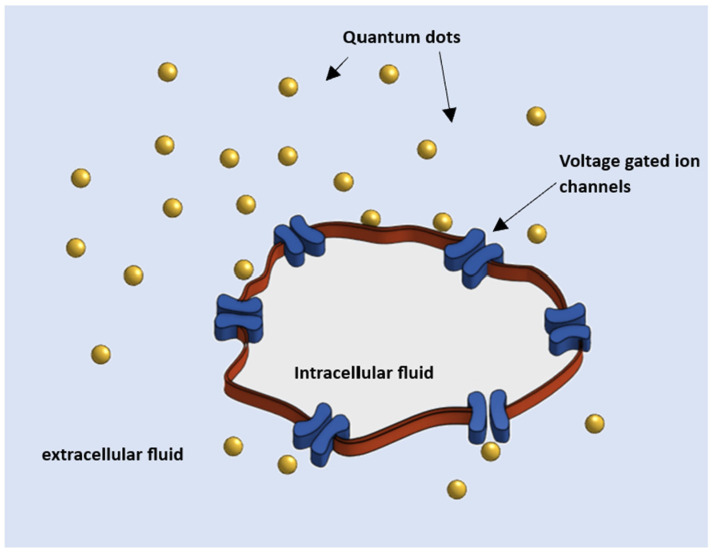
Example of an extracellular fluid containing quantum dots fabricated from materials with spontaneous polarization, suspended around a cell with voltage-gated ion channels. The cells are incubated in the extracellular fluid containing the mobile, potential-producing quantum dots.

In this article, we demonstrate that nanocrystals synthesized from materials with large, built-in spontaneous polarization produce potentials surrounding each nanocrystal and that these potentials are sufficient to switch voltage-gated ion channels when a nanocrystal is close to a voltage-gated ion channel as shown in Figure 1 This demonstration suggests that ion channel gating may be accomplished by using standard labeling techniques to bind nanostructures to ion channels or by incubating cells containing voltage-gated ion channels in a colloidal suspension of nanocrystals with built-in spontaneous polarizations.

As discussed previously in this article, we are considering the gating of voltage-gated ion channels in close proximity to nanocrystals that have built-in spontaneous polarization. This approach eliminates the need for the meticulous and precise placement of an electrode near the ion channels of cells, which is generally accomplished in situ for cells immobilized on a substrate. Such an electrode is exemplified by the electrode used in a patch-clamp apparatus [1] integrated with an optical microscope. The use of colloidal nanocrystals surrounded by potential fields sufficient to gate ion channels eliminates the need for the precise placement of an electrode near an ion channel while the cell is on the observation stage of an optical microscope. Voltage-gated ion channels are ubiquitous in the membranes of many cells. In such cells, we find an electrolyte containing a variety of ions of different concentrations in the extracellular region, and an electrolyte in the cytoplasmic interior of the cell with ionic concentrations that are different from those in the extracellular region It is well known that the Nernst equation determines the built-in potential difference across the cell membrane and, therefore, the ion channel as well [1]. This potential is expressed in terms of the logarithm of the ratio of ionic concentration in the extracellular region to the ionic concentration in the cytoplasmic interior of the cell.

Voltage-gated ion channels in a cell membrane open and close in response to the electric potential around them. This gating process requires potentials of a few mV. For voltage-gated ion channels, it is known that a potential difference of about 6 mV over the 7 nm thickness of an ion channel embedded in the cell membrane is sufficient. When such a potential—in our case, a potential that emanates from a nanocrystal (quantum dot) made of a material with an intrinsic spontaneous polarization—is applied to the ion channel, it undergoes a conformational change that allows ions which are present in the electrolyte to diffuse through the ion channel; this latter process defines the open state of a voltage-gated ion channel [1]. Herein, we demonstrate that nanocrystals with built-in spontaneous polarizations may produce electric potential large enough to cause such physiological effects. Several factors have recently come into play that stimulate a more detailed analysis of this possibility. First, it has recently been noted that effective spontaneous polarization increases in line with decreasing quantum dot diameter [7,8]. Second, there has been interest in using SiC—which has a built-in spontaneous polarization—for biological modulation [9]. Motived by these factors, as well as by the clear advantages of eliminating an external electrode and the need for precise placement of an external electrode, typically while a cell is under observation on the stage of an optical microscope, the present paper investigates theoretically the range of potential fields produced in aqueous environments in the vicinity of ZnO, GaN, and SiC quantum dots with built-in spontaneous polarizations. It is found that the electric potential produced in the proximity of quantum dots fabricated from materials that have a spontaneous polarization decreases with an increase in the radius of the quantum dot, and with an increase in the distance from the center of the quantum dot to an arbitrary point at a distance r. The present paper also analyzes the effect of Debye screening on the electric potential produced by quantum dots fabricated from selected materials with spontaneous polarization.

## 2. Materials and Method

Materials that have internal spontaneous polarization exhibit large uniform polarization in the volume of the quantum dot as well as surface charge on the surface enclosing the quantum dot. It is well known that the potential of a structure of volume, V, and surface area, S, that have an internal polarization, P, is denoted by the produced surface charge, σ, and a volume charge, ρ [10]. This can be represented in terms of the unit normal to the surface, n, and the polarization, P, given by σ=P ⋅ n and ρ = −div(P).

In this case, the volume charge, ρ, vanishes since P is constant, and the potential is determined entirely by σ. This is given by

σ = Pcosθ,(1)

where θ is the angle with respect to the c-axis of the semiconductor crystal. For a surface normal to P, such as a surface normal to the c-axis of a wurtzite material, the electric field produced on a planar surface is given by σ divided by the dielectric constant in the region bounding the surface of the spontaneously polarized material. As we shall discuss below, for GaN, experimental values and surface charge [11] range from −0.018 to 0.023 C/m^2^, respectively. Dividing these values of charge per unit area by the dielectric constant of free space, we obtain electric fields of $-2.03\;\times \;10^{9}$ V/m and $-2.60\;\times \;10^{9}$ V/m. These are extremely large electric fields. For GaN quantum dots embedded in AlN, these fields are reduced by an order of magnitude [12] and, as we shall discuss, for quantum dots embedded in water, these fields are reduced by almost two orders of magnitude since the dielectric constant of water is about 80. In addition, as we shall soon discuss, these fields are further reduced via Debye screening in cases where the quantum dots are in an electrolyte, as is the case for biological media.

From (1), the surface charge in the upper hemisphere of the quantum dot is positive and ranges from a positive maximum at the pole to zero at the equator, and is negative in the lower hemisphere, where it ranges from zero at the equator to a negative maximum at the lower pole. Such distribution is reminiscent of a dipole and is azimuthally symmetric. Therefore, a surface charge in a quantum dot of radius a is equivalent to charge Q located at s/2 along the c-axis, as well as charge –Q located at –s/2 along the c-axis; in other words, the potential produced at a distance by such a structure is that of a dipole with a dipole moment given by Qs:

(2) $$V=\left(\frac{Q}{4\pi \epsilon_{o}\epsilon_{r}}\right)\left(\frac{1}{r_{b}}-\frac{1}{r_{a}}\right),$$

where ${r_{b}}^{2}=r^{2}+{\left(\frac{s}{2}\right)}^{2}-\;\mathrm{rscos}\theta \;$and ${r_{a}}^{2}=r^{2}+{\;(\frac{s}{2})}^{2}+\mathrm{rscos}\theta \;$with $\frac{s}{2}=\frac{2a}{\pi }$ and $-\frac{s}{2}=-\frac{2a}{\pi }$.

This formalism effectively captures the fundamental dipole nature of the surface charge distribution, which is crucial for accurately describing the potential in proximity to the surface of the quantum dot. It is important to note that this proximity refers to a distance greater than the separation between the two charges, +Q and −Q, while also considering that this distance does not tend toward infinity. This particular scenario is taken into consideration with regard to the stochastic suspension of spontaneously polarized quantum dots surrounding a cell submerged in a water-based electrolyte.

(3) $$Q=P\pi a^{2}$$

where a is the radius of quantum dot.

Considering θ=0, it follows that along the c-axis, the electric potential has its maximum value, given by

(4) $$V=\left(\frac{Q}{4\pi \epsilon_{o}\epsilon_{r}}\right)\left(\frac{1}{\left(r\;-\frac{s}{2}\right)}-\frac{1}{\left(r+\frac{s}{2}\right)}\right),$$

where $\epsilon_{o}$ is the dielectric constant of free space and $\epsilon_{r}\;$is the relative dielectric constant of the medium.

For quantum dots immersed in a water-based ionic electrolyte—as in physiological environments—it is essential to include the effects of Debye screening outside of the quantum dot. It is well known that Debye screening in an electrolyte causes the potential to fall off exponentially; in (5) and (6), the 1/e-length is the Debye length, which is given explicitly in its standard form in (7), it is a function of the electrolyte concentration. Because of this Debye screening and the dielectric constant of the water, the potential along the c-axis is given by

(5) $$V=\left(\frac{Q}{4\pi \epsilon_{o}\epsilon_{r}}\right)\left(\frac{1}{\left(r\;-\frac{2a}{\pi }\right)}-\frac{1}{\left(r+\frac{2a}{\pi }\right)}\right)e^{[-(r\;-\;a)/\lambda_{D}]}$$

Combining these results, it follows that

(6) $$V=\left(\frac{a^{3}P}{\pi \epsilon_{o}\epsilon_{r}}\right)\left(\frac{1}{\left(r^{2\;}-{\left(\frac{2a}{\pi }\right)}^{2}\right)}\right)e^{[-(r\;-\;a)/\lambda_{D}]}$$

The spontaneous polarization, P, in the expression for V may be expressed in Coulombs per square meter since the induced surface charge on a surface is given by σ=P ⋅ n and its magnitude has been studied in several semiconductors [13]. Among the materials manifesting a spontaneous polarization, ZnO, GaN, and 2H-SiC exhibit relatively high values. Recently calculated values of P for ZnO, GaN, and 2H-SiC are −0.050, −0.020, and −0.029 C/m^2^, respectively [8]. These values are in general agreement with previously reported values of −0.057 C/m^2^ [14], −0.018 to −0.023 C/m^2^, [11] and −0.0111 to −0.0432 C/m^2^ [15] for ZnO, GaN, and 2H-SiC, respectively.

**Figure 2:**
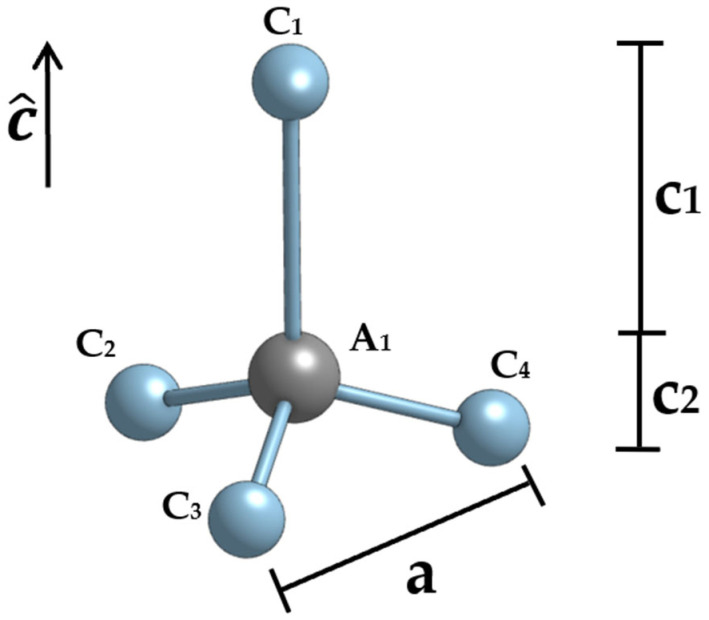
The c1 and c2 components of the unit cell structure for wurtzite materials.

Importantly, in [7], the spontaneous polarization for quantum dots composed of these materials is calculated by summing the polarizations of the c1 and c2 components of the unit cell structure for wurtzite materials, as shown in Figure 2.

As the quantum dots become larger upon stacking c1 and c2 units successively to increase the quantum dot diameter, the values of P alternate between P(high) and P(low), as discussed previously [11,12], forming two separate curves for P which merge for >50 layers to a common asymptotic value. The values of the spontaneous polarizations are shown in Table 1 for ZnO, GaN, and 2H-SiC thicknesses of 4 layers, 8 layers, 15 layers, and 22 layers, respectively.

The values in Table 1 are obtained using the method described in [4], in which the net polarizations are determined by adding the polarization of each new monolayer to the polarization of the underlying layers. For the smaller quantum dots, the values of P alternate above and below the asymptotic value and associated bulk value and, importantly, can be significantly larger than the value of P for a given semiconductor with built-in spontaneous polarization. The saturation of the quantum dot polarization as a function of the number of monolayers occurs rapidly for smaller quantum dots since each successive layer represents a major perturbation for the underlying layers, but as the quantum dots become larger, the saturation is slower since the polarization of each successive added layer contributes a diminishing amount to the net polarization of the quantum dot. The possible roughness of the nearly spherical quantum dots decreases as the radius increases.

Taking the P(low) values for ZnO, GaN, and SiC for quantum dots with 5 nm radii, their potential values at r = a are 302 mV, 246 mV, and 276 mV, respectively; in determining these values, the relative dielectric constant of the water surrounding the quantum dots has been taken to be 80. In addition, the potential equation gives the values along the c-axis as noted previously. Of course, at the opposite pole of the quantum dot, the sign of the potential changes, and in the equatorial plane, the potential is zero. These potentials are sufficient to gate a voltage-gated ion channel when they are dropped over the thickness of the ion channel, since a potential of 6 mV is known to be sufficient for such processes [16]. While these large spontaneous polarizations for small quantum dots—5 nm radius—lead to significant potentials at the quantum dot surface, it is important to consider that in addition to the continuing fall-off of the Coulomb potential from dipole, Qs, for r > a, there is an additional fall-off due to Debye screening. For distances from the quantum dot surface that are smaller compared to the quantum dot radius, the Debye fall-off contributes another factor of $e^{-(r\;-\;a)/\lambda_{D}]}$. For a monovalent ion concentration, the Debye length at room temperature is given by

(7) $$\lambda_{D}=9.62\;\mathrm{nm}/\sqrt{c},$$

where c is the ionic concentration of the electrolyte surrounding the quantum dot measured in moles per cubic meter, and 1 mole/liter equals 1000 moles per cubic meter.

For reference, in the typical physiological concentration of 100 moles per cubic meter, which equals 0.1 mole per liter or 100 mM, $\lambda_{D}$ = 0.962 nm or about 1 nm. In one, two, and three Debye lengths, the Debye screening causes an additional reduction in the potential by a factor of approximately 0.368, 0.135, and 0.050, respectively.

## 3. Results

Using the spontaneous polarization values shown in Table 1 above, we calculate the electric potential produced in the vicinity of the quantum dots using (6). However, to choose between P(high) and P(low) from Table 1, one requires knowledge of the exact diameter of the quantum dot to within one monolayer. When the exact diameter is known, one may use the corresponding polarization as given in [7,8]. To estimate the spontaneous polarization and the potential produced by the quantum dot in the case where the exact diameter is not known, one may use the average value of the spontaneous polarization and, accordingly, in the following calculations, we use the average spontaneous polarization value, which is the average of P(high) and P(low).

In this analysis, the distances from the surface of a quantum dot to an arbitrary point, r−a, are taken as 0.5 nm and 1 nm in order to assess the ability of the quantum dot to create a physiological effect. It is at these distances that electric potential produced around the quantum dot is of the magnitude to create a physiological effect. To analyze the effect of Debye screening on the produced electric field, we vary the values of Debye length from 0.33 nm to 2.33 nm. These values of Debye length were chosen since they are representative of the Debye lengths of interest in physiological environments [17,18,19,20].

**Figure 3:**
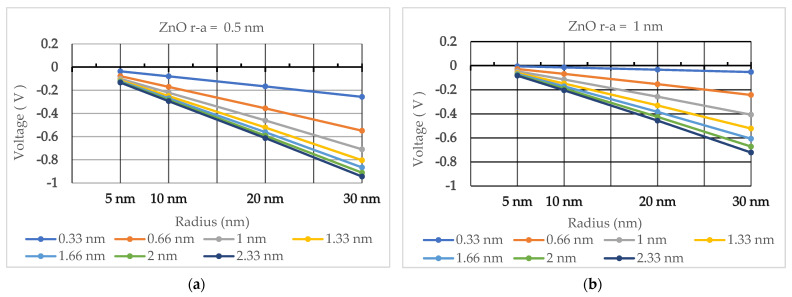
The calculated electric potential for ZnO quantum dots with radii from 5 nm to 30 nm for Debye lengths ranging between 0.33 nm and 2.33 nm. (**a**) Electric potential calculated for r−a=0.5 nm. (**b**) Electric potential calculated for r−a=1 nm.

**Figure 4:**
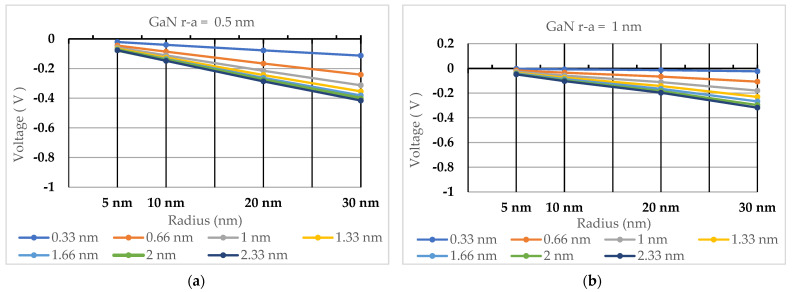
The calculated electric potential for GaN quantum dots with radii from 5 nm to 30 nm for Debye lengths ranging between 0.33 nm and 2.33 nm. (**a**) Electric potential calculated for r−a=0.5 nm (**b**) Electric potential calculated forr−a=1 nm.

**Figure 5:**
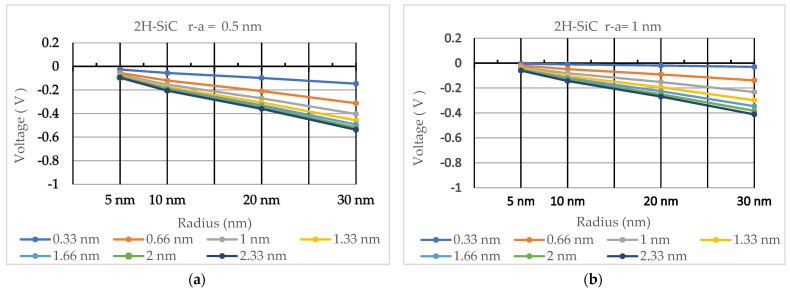
The calculated electric potential for 2H-SiC quantum dots with radii from 5 nm to 30 nm for Debye lengths ranging between 0.33 nm and 2.33 nm. (**a**) Electric potential calculated for r−a=0.5 nm. (**b**) Electric potential calculated for r−a=1 nm.

The values of electric potential (in volts) are shown in Table 2, Table 3, Table 4, Table 5, Table 6 and Table 7 for ZnO, GaN, and 2H-SiC quantum dots of 5 nm, 10 nm, 20 nm, and 30 nm radii. Electric potential trends as a function of quantum dot radius and Debye length are illustrated in Figure 3, Figure 4 and Figure 5.

## 4. Discussion

The use of electrically charged nanostructures to control ion channels has been previously demonstrated in the case where a laser field is used to polarize quantum dots that do not have a built-in spontaneous polarization [21]. The case considered in the present paper is more of general utility since it relies on the use of quantum dots with internal spontaneous polarization and does not require an external laser. Previous experiments [21] where quantum dots were polarized with an external laser established the viability of using a colloidal suspension of nanocrystals—quantum dots—to gate voltage-gated ion channels. In these previous experiments, the nanocrystals were polarized via the external laser field. In this article, it has been demonstrated that nanocrystals with built-in spontaneous polarization produce potential sufficient to gate voltage-gated ion channels without the use of an external laser.

One can conclude from the above results that the electric potential produced around quantum dots composed of materials with spontaneous polarization decreases with an increase in their radius and an increase in the Debye screening length.

Quantum dots made of ZnO produce the lowest electric potential among the three materials examined in this paper, followed by quantum dots made of 2H-SiC. Quantum dots made of GaN produce the highest electric potential, as high as −3.62191 mV for a 5 nm GaN quantum dot at a Debye length of 0.33 nm.

Furthermore, it should be emphasized that these findings are based on the use of quantum dots with intrinsic spontaneous polarization rather than quantum dots polarized through the application of external fields.

As emphasized previously, spontaneous polarization is a type of inherent polarization in some materials, in which they naturally have an electric field due to an asymmetric charge distribution within the material. In addition to the physiological applications of spontaneous polarization that we have discussed in this paper, there are many other applications. In semiconductor devices, this effect can be used for the manipulation of charge carriers and the creation of 2D electron/hole gases [22], random-access memory [23], sensors [24], and radio-frequency identification. Furthermore, these materials are used in many other devices in which this effect may be non-desirable, such as many types of semiconducting electronic and photon devices. The phenomenon of spontaneous polarization has been discussed in non-physiological applications [25,26]. In addition, spontaneous polarization has been discussed for a wide variety of materials, including CdS [27], ZnO [27,28], Fe-based compounds [29], metal-free molecular ferroelectrics [30], LiNbO 3 [31], BiFeO 3 [32], BN [33], and GaN [11].

## 5. Conclusions

Due to their inherent spontaneous polarization, ZnO, GaN, and SiC quantum dots exhibit an electric potential as a function of the radius along the c-axis of wurtzite crystals. An equation is formulated to evaluate this electrical potential while considering the Debye screening effect around the quantum dot. For the ZnO, GaN, and SiC quantum dots immersed in typical physiological electrolytes, it is found that the large potential generated by the spontaneous polarization of the quantum dots exceeds the potential needed to gate voltage-gated ion channels.

## Figures and Tables

**Table 1:** The spontaneous polarization values for ZnO, GaN, and 2H-SiC of radii of 5 nm, 10 nm, 20 nm, and 30 nm.

Radius of Quantum Dot a	ZnO P(high) C/m^2^	ZnO P(low) C/m^2^	GaN P(high) C/m^2^	GaN P(low) C/m^2^	2H-SiC P(high) C/m^2^	2H-SiC P(low) C/m^2^
5 nm	−0.039	−0.080	−0.004	−0.065	−0.012	−0.073
10 nm	−0.044	−0.069	−0.011	−0.046	−0.020	−0.059
20 nm	−0.047	−0.062	−0.016	−0.031	−0.024	−0.040
30 nm	−0.049	−0.060	−0.019	−0.029	−0.028	−0.034

**Table 2:** Electric potential produced by ZnO quantum dots at r−a=0.5 nm.

Debye Length $\lambda_{D}$	0.33 nm	0.66 nm	1 nm	1.33 nm	1.66 nm	2 nm	2.33 nm
Radius of Quantum Dot a							
5 nm	−0.03655	−0.07796	−0.10087	−0.11419	−0.12305	−0.12952	−0.13419
10 nm	−0.08012	−0.17091	−0.22112	−0.25033	−0.26975	−0.28393	−0.29416
20 nm	−0.16701	−0.35624	−0.46090	−0.52178	−0.56227	−0.59181	−0.61314
30 nm	−0.25730	−0.54884	−0.71009	−0.80388	−0.86626	−0.91178	−0.94463

**Table 3:** Electric potential produced by ZnO quantum dots at r−a=1 nm.

Debye Length $\lambda_{D}$	0.33 nm	0.66 nm	1 nm	1.33 nm	1.66 nm	2 nm	2.33 nm
Radius of Quantum Dot a							
5 nm	−6.2465 × 10^−3^	−0.02842	−0.04758	−0.06097	−0.07080	−0.07844	−0.08419
10 nm	−0.01526	−0.06941	−0.11619	−0.14891	−0.17292	−0.19157	−0.20563
20 nm	−0.03397	−0.15458	−0.25874	−0.33160	−0.38506	−0.42659	−0.45789
30 nm	−0.05363	−0.24402	−0.40846	−0.52349	−0.60789	−0.67344	−0.72286

**Table 4:** Electric potential produced by GaN quantum dots at r−a=0.5 nm.

Debye Length $\lambda_{D}$	0.33 nm	0.66 nm	1 nm	1.33 nm	1.66 nm	2 nm	2.33 nm
Radius of Quantum Dot a							
5 nm	−0.02119	−0.04521	−0.05849	−0.06621	−0.07135	−0.07510	−0.07781
10 nm	−0.04042	−0.08621	−0.11154	−0.12627	−0.13607	−0.14322	−0.14838
20 nm	−0.07815	−0.16669	−0.21567	−0.24415	−0.26310	−0.27692	−0.286902
30 nm	−0.11331	−0.24169	−0.31270	−0.35400	−0.38147	−0.40151	−0.41598

**Table 5:** Electric potential produced by GaN quantum dots at r−a=1 nm.

Debye Length $\lambda_{D}$	0.33 nm	0.66 nm	1 nm	1.33 nm	1.66 nm	2 nm	2.33 nm
Radius of Quantum Dot a							
5 nm	−3.62191 × 10^−3^	−0.01648	−0.02759	−0.03535	−0.04105	−0.04548	−0.04882
10 nm	−7.69523 × 10^−3^	−0.03501	−0.05861	−0.07512	−0.08723	−0.09663	−0.10372
20 nm	−0.01465	−0.06665	−0.11157	−0.14299	−0.16604	−0.18394	−0.19744
30 nm	−0.02362	−0.10746	−0.17987	−0.23053	−0.26769	−0.29656	−0.31832

**Table 6:** Electric potential produced by 2H-SiC quantum dots at r−a=0.5 nm.

Debye Length $\lambda_{D}$	0.33 nm	0.66 nm	1 nm	1.33 nm	1.66 nm	2 nm	2.33 nm
Radius of Quantum Dot a							
5 nm	−0.02610	−0.05569	−0.07205	−0.08157	−0.08790	−0.09251	−0.09585
10 nm	−0.05602	−0.11949	−0.15459	−0.17500	−0.18859	−0.19849	−0.20565
20 nm	−0.09805	−0.20917	−0.27062	−0.30637	−0.33014	−0.34748	−0.36000
30 nm	−0.1463	−0.3121	−0.40390	−0.45725	−0.49273	−0.51862	−0.53731

**Table 7:** Electric potential produced by 2H-SiC quantum dots at r−a=1 nm.

Debye Length $\lambda_{D}$	0.33 nm	0.66 nm	1 nm	1.33 nm	1.66 nm	2 nm	2.33 nm
Radius of Quantum Dot a							
5 nm	−4.46 × 10^−3^	−0.02030	−0.03398	−0.04355	−0.05057	−0.056027	−0.06013
10 nm	−0.01066	−0.04852	−0.08123	−0.104107	−0.12089	−0.133927	−0.143755
20 nm	−0.019946	−0.09075	−0.15192	−0.194703	−0.22609	−0.250474	−0.268854
30 nm	−0.030504	−0.13880	−0.23233	−0.2977658	−0.34577	−0.383058	−0.411168

## Data Availability

All data used and/or analyzed during the current study are shown within the study. If further data are required, they can be made available to the corresponding author upon reasonable request.
